# Consequences of the natural course of articular osteochondrosis in pigs for the suitability of computed tomography as a screening tool

**DOI:** 10.1186/s12917-014-0212-9

**Published:** 2014-09-09

**Authors:** Kristin Olstad, Jørgen Kongsro, Eli Grindflek, Nils I Dolvik

**Affiliations:** Department of Companion Animal Clinical Sciences, Faculty of Veterinary Medicine and Biosciences, Norwegian University of Life Sciences, Oslo, Norway; Research and Development, Norsvin, Hamar Norway

**Keywords:** Articular osteochondrosis, Leg weakness, Pig, CT, Histology, Development, Screening, Selection, Breeding

## Abstract

**Background:**

A significant heritability has been documented for articular osteochondrosis. Selection against osteochondrosis has historically been based on macroscopic evaluation, but as computed tomography (CT) now is used to select boars with optimal body composition it can potentially also be used to screen for osteochondrosis. False negative diagnosis will occur if defects have not developed or have resolved prior to screening at a single time point.

The aim of the current study was to assess the suitability of the use of CT at a single point in time as a screening tool in piglets for articular osteochondrosis, which is known to be a highly dynamic condition in which lesions develop and resolve over time.

**Methods:**

Male Landrace piglets (*n* = 18) were serial CT scanned from 2–8 times at biweekly intervals from 70–180 days of age. At each interval, 1–2 piglets were euthanased and the left distal femur processed for histological validation.

**Results:**

A total of 795 defects were identified in the 112 available CT scans. Within the hind and fore limbs, the incidence of defects was highest in the stifle (*n* = 321) and elbow joints (*n* = 110), respectively. Ninety-eight per cent of the defects in the stifle and elbow joints had developed by the 7^th^ examination interval when the piglets were a mean age of 159 days old. The proportion of defects that resolved was lowest in the stifle joint at 51% and highest in the elbow joint at 69%.

**Conclusions:**

Scanning of the current piglets at an age of 159 days resulted in detection of 98% of the total number of defects that developed up to the maximum age of 180 days. The proportion of defects that resolved ranged from 51–69% for different joints, but may not adversely affect prevalence as this category of false negative diagnosis will result in selection of pigs that are disposed for healing. Optimally timed CT is a powerful screening tool for osteochondrosis.

## Background

Osteochondrosis is the most common of the developmental orthopaedic diseases and affects several animal species including pigs, horses, cattle and dogs [1,2]. It is defined as a focal delay in enchondral ossification and can develop both in the metaphyseal growth plate (physeal osteochondrosis) and the sub-articular epiphyseal growth cartilage (articular osteochondrosis) [2]. Articular osteochondrosis often occurs multi-focally at predilection sites in more than one joint simultaneously [3–6]. It can progress to cartilage fracture and the formation of intra-articular fragments known as *osteochondrosis dissecans* (OCD), which condition has been associated with “leg weakness” in pigs [7] and lameness in horses [6]. Hence, osteochondrosis and OCD can compromise the health, welfare and athletic or reproductive performance of affected individuals [6–12]. A significant heritability has been documented in pigs [13,14] and horses [15,16]. Screening for and selection against osteochondrosis is therefore a mandatory part of sustainable management of species with high prevalence of the disease.

In pigs, selection against osteochondrosis has historically been based on macroscopic evaluation of related slaughtered individuals [3,4] and estimation of a phenotypic breeding value. In horses, breeding values are estimated based on radiographic evaluation [17,18]. Computed tomography has proven equal to manual dissection for quantification of lean tissue in pigs [19–21], has practical advantages over MRI [22,23] and is therefore used to select boars with optimal body composition in some countries [24,25]. An effect of body weight on the prediction of protein has, however, been demonstrated, meaning that CT quantification must be standardised to occur within a limited weight window [26]. All stages of the pathogenesis of osteochondrosis from vascular failure via ischaemic chondronecrosis to a focal delay in enchondral ossification were detectable or could be inferred in previous ex vivo arterial contrast-enhanced micro-CT or contrast-enhanced conventional CT scans of naturally occurring and experimentally induced osteochondrosis lesions in foals [27,28]. In a related study the positive predictive values of CT and macroscopic evaluation for the diagnosis of osteochondrosis in the distal femur of piglets were 100% and 80%, respectively [29]. The accuracy of selection can therefore be improved by advancing from macroscopic to CT radiographic evaluation of osteochondrosis in pigs.

Irrespective of technique, the principal risk when screening for osteochondrosis is a false negative diagnosis because true positive, genetically predisposed individuals may be included in the breeding stock and selection strategies will fail to reduce prevalence of the disease. The risk is particularly high in the case of osteochondrosis as it may only be feasible to screen at a single point in time, and the disease is known to be highly dynamic. For example, observational and experimental studies in piglets [30–32] and foals [33–35] have confirmed that the first step in the pathogenesis of osteochondrosis is focal failure of the blood supply to epiphyseal growth cartilage. Failure of the blood supply leads to ischaemic necrosis of susceptible chondrocytes at intermediate depth of the growth cartilage. Areas of ischaemic chondronecrosis will not convert into bone and cause the focal delay in enchondral ossification that is characteristic of osteochondrosis [2]. The blood supply runs within cartilage canals that are present temporarily in the early phases of growth. The age windows have been documented in the elbow [36,37] and distal femur [30,38] of different groups of piglets. From subsequent studies in one group of foals, it is clear that the windows vary between joints, and between regions within joints [34,39,40]. False negative diagnosis may occur if an individual is screened before lesions have developed, but this can be avoided by screening after the age when all developed lesions have become detectable. Determination of an optimal screening age will, however, depend upon access to species-specific information on the age when the blood supply disappears and the growth cartilage becomes resistant to ischaemia at each predilection site.

Also, spontaneous regression or reversion to normal appearance has been observed when osteochondrosis or osteochondral abnormalities were monitored longitudinally by plain, planar radiography in horses [17,18]. The proportion of defects that regressed varied between different joints [17,18]. Signs of repair or healing have been noted in macroscopic studies in pigs [3–5,41], but the proportion of defects that resolve has not been quantified. False negative diagnosis may therefore occur as well if a defect has resolved prior to screening at a single time point. It may not be possible to eliminate this type of false negative diagnosis, but the proportion of defects that resolve should be quantified so that it may be factored in, for example by weighting the scores for different joints, when considering the application and efficacy of different selection strategies.

The aim of the current study was to assess the suitability of the use of CT at a single point in time as a screening tool in piglets for articular osteochondrosis, which is known to be a highly dynamic condition in which lesions develop and resolve over time.

## Methods

Animal use and study design were approved by the National Animal Research Authority. All animals were kept in accordance with the national legislation (Animal Welfare Act 2009-06-19-97; Regulation for the keeping of pigs in Norway 2003-02-18-175).

The study group consisted of 18 male Landrace piglets that had been purpose-bred for the study by artificial insemination. All piglets were sired by a boar with a high risk of osteochondrosis among his offspring according to phenotypic estimated breeding value. The piglets were born to 8 different sows that were selected because their predicted farrowing dates fitted with the study start date.

The CT unit is part of the Norwegian Pig Breeder Association Norsvin’s central boar performance test station. Male piglets were therefore recruited from the litters of the 8 sows whilst still on-farm. The lower age limit was determined by the consideration that piglets should weigh ≥ 20 kg to ensure sufficient immuno-competence before entering the CT unit. Piglets were vaccinated against *Actinobacillus pleuropneumoniae* (Porcilis APP, Intervet/MDD Animal Health, Boxmeer, the Netherlands) on admittance to the CT unit 7–8 days before the start of the study and boosted 3 weeks later. The upper age limit of the piglets was determined by the fact that body composition currently is evaluated at 120 kg body weight.

On the study start date, the piglets were a mean of 75 days old (range: 70–82, Table 1) and weighed a mean of 26 kg (range: 20–38 kg, Table 1).

**Table 1:** **Piglet age and weight, number of femurs processed for histological validation and available CT scans**

**Interval**	**1** ^**st**^	**2** ^**nd**^	**3** ^**rd**^	**4** ^**th**^	**5** ^**th**^	**6** ^**th**^	**7** ^**th**^	**8** ^**th**^
Mean piglet age in days (range: −5/+7 days)	75	89	103	117	131	145	159	173
Mean piglet weight in kg	26	36	46	56	68	83	98	116
Weight range in kg	20-38	27-46	36-57	44-71	54-82	66-96	79-112	96-128
Number of left distal femurs processed for histological validation	0	1	1	2	2	2	2	7*
Number of piglets scanned	18	18	17	16	14	12	10	7*
Cumulative number of scans available	18	36	53	69	83	95	105	112

*The piglet identified as piglet number 11 in Table 2 constituted a drop-out from histological validation at the 8^th^ interval.

### Study design

The study design consisted of serial CT scanning of individual piglets from 2–8 times at biweekly intervals (Table 2).

**Table 2:** **Summary of the number of times that defects developed and resolved radiographically, per piglet**

**Piglet number**	**Number of times scanned**	**Number of defects present at 1** ^**st**^ **interval; time of development unknown**	**Number of defects that developed from the 2** ^**nd**^ **-8** ^**th**^ **interval**	**Number of times that defects were characterised as persisting**	**Number of defects that resolved**	**Sum of defects present at 1** ^**st**^ **interval and defects that developed**	**Proportion of defects that resolved**
1	2	12	4	6	4	16	25%
2	3	9	8	17	2	17	12%
3	4	17	32	40	25	49	51%
4	4	25	22	60	20	47	43%
5	5	13	28	39	18	41	44%
6	5	17	26	43	27	43	63%
7	6	9	21	41	16	30	53%
8	6	16	31	52	28	47	60%
9	7	18	13	35	23	31	74%
10	7	20	28	84	27	48	56%
11*	7	10	33	79	20	43	47%
12	8	6	57	92	26	63	41%
13	8	15	27	95	23	42	55%
14	8	10	43	101	33	53	62%
15	8	15	36	116	28	51	55%
16	8	21	54	100	58	75	77%
17	8	14	47	88	43	61	70%
18	8	6	32	34	24	38	63%
Sum	112	253	542	1122	445	795	
Mean		14	30	62	25	44	
Range		6-25	4-57	6-116	2-58	16-75	12-77%

*Piglet 11 constituted a drop-out from histological validation at the 8^th^ interval.

At the 1^st^ interval, all piglets were anaesthetised, CT-scanned and recovered. Anaesthesia initially consisted of intra-muscular injection of 2 mg/kg midazolam (Midazolam B, Braun, Melsungen, Germany) and 10 mg/kg ketamine (Ketalar, Pfizer Inc., New York, USA). From the 3^rd^ interval, anaesthesia was changed to 2.2 mg/kg xylazine (Rompun vet, Bayer Animal Health GmbH, Leverkusen, Germany) and 4 mg/kg tiletamine/zolazepam (Zoletil Forte Vet, Virbac, Carros, France) to minimise injection volume.

From the 2^nd^ interval, 1–2 piglets were euthanased, CT-scanned and the left distal femur processed for histological validation (Table 1). Euthanasia was by captive bolt stunning and exsanguination. At the 8^th^ and final interval, all remaining piglets were euthanased, CT-scanned and the left distal femur processed for histological validation.

### CT scanning

Piglets were positioned in sternal recumbency on the patient table of a 32-slice helical CT (GE Light Speed Pro 32, GE Healthcare, Little Chalfont, UK). The front and hind limbs were extended and scanned with a fixed kV of 120, a dynamic mA of up to 650 and a slice thickness of 0.625 mm.

### Evaluation of CT scans

CT scans (*n* = 112, Table 1) were transferred to an open-source PACS workstation and DICOM viewer (www.osirix-viewer.com).

Six different joints were evaluated bilaterally, comprising the shoulder, elbow, carpus, hip, stifle and tarsus, i.e. 12 joints per scan. Within the 6 different joints, 16 regions were evaluated separately comprising the glenoid, humeral head, medial humeral condyle, lateral humeral condyle, proximal radius and carpus in the forelimb, and the acetabulum, femoral head, medial femoral condyle, lateral femoral condyle, femoral medial trochlear ridge, femoral lateral trochlear ridge, patella, distal tibia, talar medial and talar lateral trochlear ridge in the hind limb.

For each piglet, one joint was viewed at a time and all available scans of that joint were evaluated consecutively. Diagnosis of a suspected osteochondrosis lesion was based on identification of a focal radiolucent defect within or immediately adjacent to the ossification front. The location of each defect was recorded in terms of bone (e.g. distal femur), epiphyseal region (e.g. medial femoral condyle) and anatomical aspect (e.g. cranial aspect). The disto-proximal height level of the defect was recorded as the number of transverse CT slices between the defect and an anatomical landmark times the slice thickness of 0.625 mm (further details and illustrations available in [29]).

Defects that became radiographically detectable during the study, i.e. from the 2^nd^ to the 8^th^ examination interval were characterised as “developing”. In this context, the word “developed” is used to refer to when defects became radiographically detectable. Defects that were present at the 1^st^ interval were considered to have developed before the age of 70–82 days old, but the time of development was otherwise unknown. When similar changes were detected in a matching location in subsequent available scans, the defect was characterised as “persisting”. When changes were no longer detectable in or near the location of a defect in the preceding scan, the defect was characterised as “resolved”.

Scans were evaluated by a single observer with 15 years’ experience in veterinary radiology. Intra-observer agreement was affected by joint (higher in simple joints) and interval (higher in late intervals). Agreement was therefore informally assessed by scoring 1 pig, scanned the maximum possible 8 times, on 2 occasions with a time gap of 1 week. This generated 313 observations with agreement on 224 occasions (71.5%).

### Histological validation

The technique for histological validation of observed radiographic changes is described in full in a separate manuscript [29] but briefly, the left distal femur was fixed in 4% formaldehyde for 48 hours before being sawed into approximately 3 mm thick slabs in the transverse plane. The slabs were decalcified in 10% EDTA and all areas displaying macroscopically visible changes were sectioned and stained with haematoxylin and eosin for histological evaluation. Diagnosis of an osteochondrosis lesion was based on identification of an area of ischaemic chondronecrosis. Radiographic and histological changes were considered to represent the same lesion when their location matched between histological sections and CT scans.

### Data analysis

Proportions of defects that underwent spontaneous resolution were compared using Pearson’s chi-square test and a significance level of P ≤ 0.05.

## Results

The piglets were assigned ascending numbers from 1–18 by age (Table 2). If 2 piglets were the same age then numbers were assigned according to ascending weight at the time of euthanasia (individual weights available in [29]). All piglets coughed and had nasal discharge at the 5^th^ examination interval presumed to represent respiratory viral infection that resolved without treatment before the 6^th^ interval. Piglet 11 was euthanased due to acute respiratory distress after the 7^th^ interval. It was not possible to preserve the skeleton and piglet 11 therefore constituted a drop-out from histological validation at the 8^th^ interval.

In the 112 CT scans there were a total of 795 defects (Table 2). Thirty-four macroscopically visible lesions were processed for histological validation from the 17 available left distal femurs.

### Histological validation

**Figure 1:**
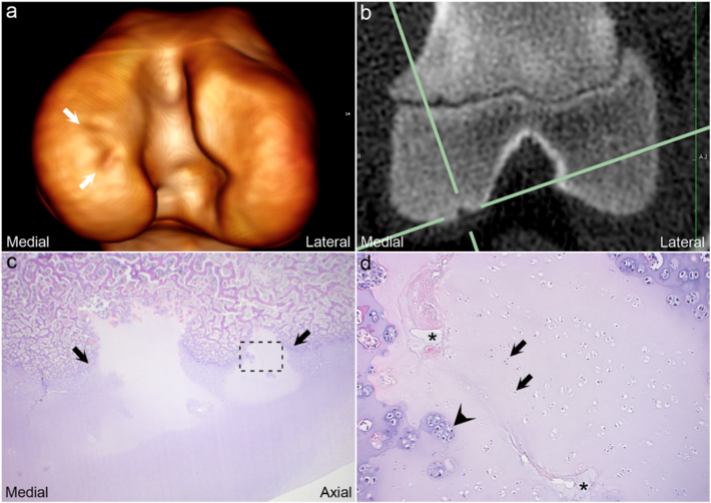
**Radiolucent defect corresponds to areas of ischaemic chondronecrosis.** Three-dimensional volume-reconstructed model **(a)**, transverse CT slice **(b)** and transverse histological section **(c, d)** from the left distal femur of piglet 6 at the 5^th^ examination interval. **(a)** There is a focal radiolucent defect within the ossification front of the medial femoral condyle (between arrows). **(b)** The defect from **(a)** is visible at the intersection of the solid green lines. **(c)** The radiolucent defect corresponds to 2 closely adjacent areas of ischaemic chondronecrosis located within the ossification front (between arrows). The stippled box indicates the location of the area that is shown at higher power magnification in **(d)**. **(d)** The figure shows a necrotic cartilage canal (asterisks) surrounded by necrotic chondrocytes (arrows), i.e. ischaemic chondronecrosis. Adjacent viable chondrocytes are grouped together several cells within a single lacuna, interpreted as proliferation (arrowhead).

Radiolucent defects corresponded to areas of ischaemic chondronecrosis in or near the ossification front in 34/34 histologically validated defects (100%, Figure 1). The cartilage superficial to all lesions was intact/unbroken, i.e. OCD was not observed.

**Figure 2:**
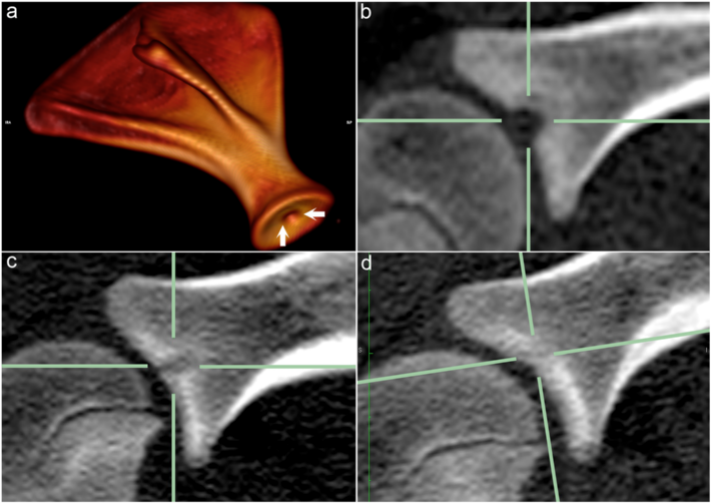
**Osseous cyst-like lesion.** Three-dimensional volume-reconstructed model **(a)** and sagittal CT slices from the right scapula of piglet 11 at the 1^st^ **(a, b)**, 3^rd^ **(c)** and 4^th^ **(d)** examination intervals. **(a)** There is a radiolucent defect in the centre of the weight-bearing surface of the glenoid (between arrows). **(b)** At the 1^st^ interval the defect is located within the ossification front (at intersection of solid green lines). **(c)** By the 3^rd^ interval the defect has become surrounded by bone and is compatible with diagnosis of an osseous cyst-like lesion. **(d)** At the 4^th^ interval the defect has become filled with mineral/bone opacity and has resolved.

**Figure 3:**
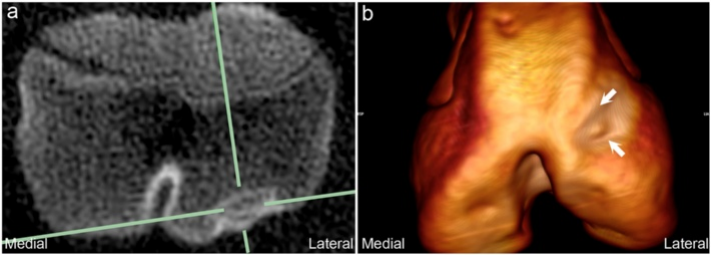
**Mixed radiopacity defect at the origin of the long digital extensor tendon.** Transverse CT slice **(a)** and 3D volume-reconstructed model **(b)** from the left distal femur of piglet 8 at the 6^th^ examination interval. **(a)** The focal, mixed radiopacity defect is visible at the intersection of the solid green lines. The subchondral bone plate is intact and the defect is surrounded by a thin rim of sclerosis on all margins to bone. **(b)** The defect is located at the origin of the long digital extensor tendon in the distal third of the femoral lateral trochlear ridge (between arrows).

The CT radiographic characteristics of the 34 examined defects were identical to the characteristics of 759/795 defects (95%). Fourteen defects (2%) were different in that, although they started as defects in the ossification front, they progressed to become extensively surrounded by bone and were then compatible with diagnosis of an osseous cyst-like lesion (Figure 2). The cyst-like lesions were observed in the shoulder, distributed as 12 defects in the humeral head and 2 defects in the glenoid. Twenty-two defects (3%) had slightly different radiographic characteristics and consisted of a focal radiolucent or mixed radiopacity defect that was located deep to a partially or fully intact subchondral bone plate and surrounded by a thin rim of sclerosis on all margins to bone (Figure 3a). These defects occurred at the origin of the long digital extensor tendon in the distal third of the femoral lateral trochlear ridge (Figure 3b) and consisted of vascularised fibrous connective tissue.

### Development of defects

A total of 253 defects were present at the 1^st^ examination interval (Table 2). This translates to 32% of defects having developed by the 1^st^ interval when the piglets were 70–82 days old and 68% of defects (542/795, Table 2) developing from the 2^nd^ to the 8^th^ interval from 84–180 days of age (Table 1).

Within the hind and fore limbs, the incidence of defects was highest in the stifle (*n* = 321) and elbow joints (*n* = 110), respectively (Table 3).

**Table 3:** **Proportion of defects undergoing spontaneous radiographic resolution for all joints and some regions, per interval**

**Joint**	**Percentage of defects that resolved per joint (445/795)**	**Region**	**Percentage of defects that resolved per region**	**Present at 1** ^**st**^ **interval**	**2** ^**nd**^ **interval, resolve/develop**	**3** ^**rd**^ **interval, resolve/develop**	**4** ^**th**^ **interval, resolve/develop**	**5** ^**th**^ **interval, resolve/develop**	**6** ^**th**^ **interval, resolve/develop**	**7** ^**th**^ **interval, resolve/develop**	**8** ^**th**^ **interval, resolve/develop**
Shoulder	56% (47/84)	(not shown)		38	9/16	9/10	9/4	8/6	5/4	6/3	1/3
Elbow	69% (76/110)			65	17/11	20/5	19/9	9/7	6/3	2/8	3/2
		Medial humeral condyle	70% (40/57)	37	12/6	3/3	12/4	7/2	4/1	2/3	0/1
		Lateral humeral condyle	64% (25/39)	21	4/1	10/2	5/3	1/5	2/2	0/4	3/1
		Proximal radius	79% (11/14)	7	1/4	7/0	2/2	1/0	0/0	0/1	0/0
Carpus	56% (53/94)	(not applicable)		19	8/11	13/19	9/12	7/11	7/17	5/2	4/3
Hip	64% (23/36)	(not shown)		11	4/5	5/6	2/5	7/2	2/5	1/0	2/2
Stifle	51% (165 /321)			72	20/36	21/43	29/47	25/36	21/53	27/26	22/7
		Medial femoral condyle	48% (73/151)	34	7/15	12/19	10/30	12/16	11/26	11/10	10/1
		Lateral femoral condyle	56% (38/68)	13	5/8	5/12	5/9	5/8	6/12	7/5	5/1
		Femoral medial trochlear ridge	35% (14/40)*	6	5/3	1/4	3/4	1/7	0/8	2/6	2/2
		Femoral lateral trochlear ridge	63% (29/46)*	16	3/9	2/6	8/2	5/1	2/5	4/4	5/3
		Patella	69% (11/16)	3	0/1	0/2	3/3	2/4	3/2	3/1	0/0
Tarsus	54% (81/150)			48	15/17	18/19	15/25	10/10	9/13	7/13	7/5
		Distal tibia	43% (22/51)	12	2/6	8/7	3/7	4/5	2/5	0/5	3/4
		Talar medial trochlear ridge	63% (34/54)	21	7/6	5/7	7/9	4/3	6/3	2/5	3/0
		Talar lateral trochlear ridge	56% (25/45)	15	6/5	5/5	5/9	2/2	1/5	5/3	1/1

*Femoral medial and lateral trochlear ridge statistically significantly different at P = 0.02.

**Figure 4:**
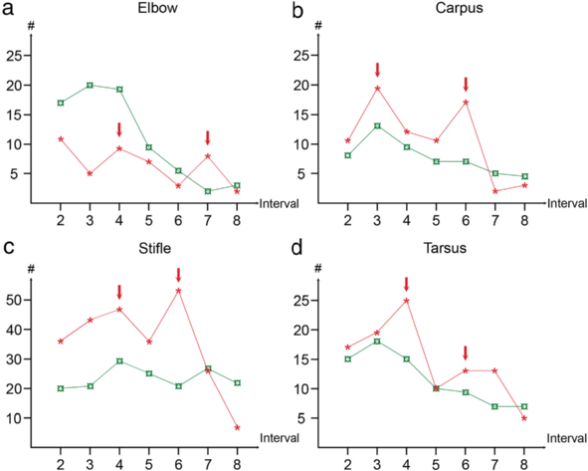
**Curves for the rate of defects developing and resolving radiographically in some joints.** The graphs show the number of defects that became visible (red asterisks, *) and resolved (green currency signs, ¤) on the y-axes from the 2^nd^ to the 8^th^ examination interval on the x-axes in the elbow **(a)**, carpus **(b)**, stifle **(c)** and tarsus **(d)**. The rate of defects developing and resolving radiographically decreased during the study. The curves for defects developing in the elbow, carpus, stifle and tarsus showed a distinct early and late peak (red arrows) as they dropped.

The rate of defects developing decreased during the observation period (Table 3, Figure 4). The rate of defects developing was zero at the 8^th^ and final interval in the proximal radius, patella and talar lateral trochlear ridge regions, but when all regions within a joint were added up the rate was not zero for any joint (Table 3). Among the forelimb joints, the rate was closest to zero in the elbow and although 3 new defects developed in both joints, the rate had remained lower for a longer time in the shoulder than in the carpus by the 8^th^ interval. In the hind limb, the rate was closest to zero in the hip, followed by the tarsus and the stifle joint at the 8^th^ interval. In the joints with highest incidence within the respective limbs, 108 of the total identified 110 defects (98%) had developed in the elbow and 314/321 defects (98%) had developed in the stifle by the 7^th^ interval (Table 3). A single new defect developed in each of the medial and lateral femoral condyle regions, whereas 5 defects developed within the femoral trochlea at the 8^th^ interval.

The curves for defects developing undulated, i.e. showed peaks and troughs as they dropped (Figure 4). A single peak value (value that was higher than the values at the preceding and subsequent intervals) was observed among the values for the shoulder joint (Table 3). In all other joints there were 2 distinct peaks, i.e. an early and a late peak.

### Resolution of defects

**Figure 5:**
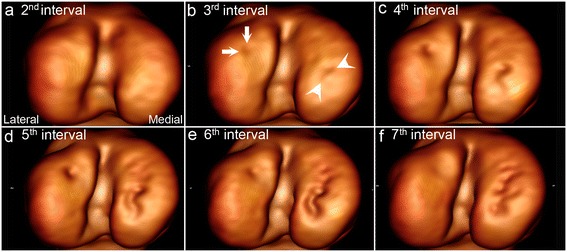
**The proportion of defects that resolved varied between regions.** Three-dimensional volume-reconstructed model of the right distal femur of piglet 17 from the 2^nd^ **(a)** to the 7^th^ **(f)** intervals. **(a)** Normal. **(b)** Defect developing in both the lateral (between arrows) and the medial (between arrowheads) femoral condyle. **(c)** Defects persisting in both the lateral and medial femoral condyle. **(d)** Defects persisting and becoming more prominently visible due to continued advancement of the ossification front within the viable tissue on the margins of the defects. **(e)** Defects persisting, but defect in the lateral femoral condyle becoming less prominently visible due to filling in with mineral/bone opacity. **(f)** Defect in the lateral femoral condyle resolved. Defect in the medial femoral condyle persisting.

Spontaneous radiographic resolution predominantly occurred by radiolucent defects becoming filled with mineral/bone opacity (Figures 2, 5). In some cases, resolution occurred by incorporation of the defect into the radiolucent line of a closely adjacent metaphyseal growth plate.

The proportion of defects that resolved was 56% (445/795) for all joints in all piglets. This was considered an underestimate because defects could have resolved in the joints of piglets that were euthanased before the maximum age of 180 days. In piglets 12–18 that were euthanased at the 8^th^ and final interval, the proportion of defects that resolved was 61% for all joints (253/383 defects, Table 2).

The proportion of defects that resolved was lowest in the stifle joint at 51% and highest in the elbow joint at 69%. The proportion of defects that resolved was higher, but not statistically significant in the medial humeral condyle (P = 0.69), lateral femoral condyle (Figure 5, P = 0.37) and talar medial trochlear ridge (P = 0.59) compared to their respective lateral and medial counterparts. The proportion of defects that resolved was statistically significantly higher at 63% in the lateral trochlear ridge of the femur compared to 35% in the femoral one (P = 0.02, Table 3).

The rate of defects undergoing resolution decreased during the study period. From 1–3 peaks were apparent among the values for defects resolving in different regions, but it was not possible to identify any common tendencies (Table 3). At the 2^nd^ interval, the rate of defects developing was higher than the rate of defects resolving in 5/6 examined joints (excluding the elbow: 85 defects developed/56 defects resolved, Table 3). By the 8^th^ interval, the relationship was reversed for 4/6 evaluated joints (excluding the shoulder and hip: 17 defects developed /36 defects resolved, Table 3). The curves for defects developing crossed the curves for defects resolving between the 6^th^ and 7^th^ intervals in the elbow and at the 7^th^ interval in the stifle, thus the rate of defects developing was lower than the rate of defects resolving from the 7^th^ interval onwards in these joints (Figures 4a, c).

### Duration of defects that resolved

In piglets 12–18 that were euthanased at the 8^th^ and final interval, 155 defects both developed and resolved during the study (Table 4). The majority of these defects (63/155 defects, 41%) persisted for 1 interval (range: 0–5 intervals, Table 4). This translates to a defect developing at one interval, persisting for a second interval before healing at the third interval, i.e. being radiographically detectable for a duration of approximately 4 weeks.

**Table 4:** **Duration of defects that developed and resolved radiographically between the ages of 84–180 days**

**Number of intervals**	**Number of defects**
0 intervals	52
1 interval	63
2 intervals	27
3 intervals	6
4 intervals	4
5 intervals	3
Sum	155

## Discussion

To the best of our knowledge the current study is the first in which articular osteochondrosis has been followed longitudinally by CT in any species. The technique has many potential applications, but the current aim was to monitor the development of osteochondrosis because the dynamic character of osteochondrosis may affect the accuracy of CT if used at a single time point as a screening tool for osteochondrosis.

### Development of defects

When considering all examined joints in all piglets, the rate of defects developing decreased during the study period. The blood supply to epiphyseal growth cartilage is only present during the early phases of growth [30,36–38] and once it has regressed, vascular failure can no longer occur. Variation was, however, detected in the decrease of defects developing between different joints, and between different regions within joints. On a joint level, this agrees with the observation that the rate of defects becoming detectable decreases at an earlier age in the fetlock and tarsus, compared to the stifle joint of horses [17,18]. Equivalent data are not available for pig joints, but radiographic closure times have been documented for all porcine metaphyseal growth plates [42]. The relationship between closure times in epiphyseal versus metaphyseal growth cartilage is not known but with exception of the hip joint, the rate of defects developing in each current studied joint decreased in an identical sequence to the sequence for radiographic closure of the metaphyseal growth plates of those joints [42]. In the current study, fewer new defects developed in the femoral condyle regions compared to the femoral trochlear regions at the 8^th^ and final interval. This agrees with the observation in horses that the blood supply regressed from the femoral condyle regions before the femoral trochlear regions [39]. In sum, the current results support that a relationship exists between disappearance of the blood supply and the age threshold for when defects develop radiographically in pigs as well as in horses. The blood supply should be studied in additional pig joints, and both the blood supply and radiographic development of defects should be studied in older pigs in the future. Within the limitations of the current study, 98% of the total number of defects that developed by the maximum age of 180 days (maximum weight: 128 kg) was detectable by the age of 159 days (mean weight: 98 kg). This level of error is similar to that which is accepted for manual dissection or CT quantification of lean tissue (~1% [19,20,22]).

### Resolution of defects

The proportion of defects that resolved varied between joints, as previously documented in horses [17,18]. Statistically significantly more defects resolved in the lateral trochlear ridge of the femur compared to the medial one. In horses, persistent osteochondrosis is more commonly observed in the lateral femoral trochlear ridge than the medial one [6]. Spontaneous healing of experimental chondral fractures in young rabbits depended on the width of the fracture pedicle, blood supply and stability of the fracture flap [43]. Whether a defect is able to heal may therefore depend on the degree to which it is rendered unstable through exposure to biomechanical load. Differences in the proportion of defects that resolved between different pig joints and joint regions may therefore be due to differences in exposure to load [4,41], and this may also explain the difference in the location of persistent osteochondrosis within the femoral trochlea between pigs and horses [6].

False negative diagnosis due to defects resolving can only be eliminated through continuous scanning which is not feasible in practice. If pigs were screened twice, it would be possible to select those individuals which, in addition to being little susceptible to the development of lesions, show a high rate of resolution of lesions and hence have a good healing tendency. In the current study, the curves for defects developing crossed the curves for defects resolving at the 7^th^ interval. The ideal dual times would therefore be early screening at 159 days of age and late screening at the time beyond 180 days of age when no new defects develop. The prevalence of osteochondrosis is so high that it may not be an option to select true negative pigs to breed from [3,7]. In mice, the ability to regenerate wounded cartilage is a heritable trait [44]. If pigs were screened twice it would be possible to select individuals that in addition to low morbidity osteochondrosis demonstrate a high proportion and may be genetically disposed for healing.

The categories of false negative diagnosis discussed in the current report apply to any kind of phenotypic screening at a single time point. The genome is constant irrespective of whether defects develop and resolve. Genomic screening may therefore supersede phenotypic screening in due course. The current results are valuable in their own right, but they are also being implemented in on-going genome-wide association studies that may enable genomic selection against osteochondrosis in the future.

In the interim before genomic screening becomes available, the 51% lowest and 69% highest proportions of resolving defects in the highest incidence stifle and elbow joints, respectively, indicate that screening will result in detection of more defects in the stifle than in the elbow at any given time. One option for addressing this difference is to introduce weighting of osteochondrosis scores in the two joints. The current results suggest that if stifle scores were weighted by a factor of 1, then elbow scores were 18% less reliable and could be weighted by a factor of 0.8 in phenotypic estimated breeding values.

### Aetiology

Peaks in the curves for the rate of defects developing imply that there are periods of particular high susceptibility to vascular failure during the age window when the blood supply is present. It was considered whether events that were common to the whole group of piglets, i.e. moving to the CT unit, vaccination and respiratory infection could have caused vascular failure. In an experimental study in the distal femur, the time lag from vascular transection to delayed ossification was 29 days [32]. The only event that occurred 2 intervals (28 days) prior to the incidence peaks in the distal femur was booster vaccination. In the absence of a satisfactory external/environmental explanation, the principal alternative is that the incidence peaks may have been precipitated by programmed internal events. The blood supply to epiphyseal growth cartilage is not identical between individuals of the same age, but it follows the same basic pattern of distribution and development [30,31,36,37]. Cartilage canals regress by chondrification and incorporation into the ossification front where canal vessels anastomose with vessels in the subchondral bone (Figure 1 and Table 2 in [31,37]. If the majority of piglets had 2 cartilage canal vessels left to incorporate between the ages of 84–180 days, this could explain why there were 2 peaks in the curves for the rate of defects developing in most joints. The current results then further support the suggestion that cartilage canal vessels are particularly vulnerable to failure during the process of incorporation into the ossification front [31,34,37].

### Limitations

The principal limitations of the current study were that defects were only histologically validated in the distal femur, and that piglets were not followed beyond 180 days of age. Both of these avenues may be pursued further in the future.

## Conclusions

Scanning of the current piglets at an age of 159 days (mean weight: 98 kg) resulted in detection of 98% of the total number of defects that developed up to the maximum age of 180 days (maximum weight: 128 kg). The proportion of defects that resolved ranged from 51–69% for different joints, but may not adversely affect prevalence as this category of false negative diagnosis will result in selection of pigs that are disposed for healing. Combined with the documented positive predictive value of CT for the diagnosis of osteochondrosis of 100%, optimally timed CT is the most powerful currently available screening tool for osteochondrosis.
